# Supplementary figures and images for: Genomics versus mtDNA for resolving stock structure in the silky shark (Carcharhinus falciformis)
Source: PeerJ. 2020 Oct 21;8:e10186. doi: 10.7717/peerj.10186 (PMC7585369; doi:10.7717/peerj.10186)

Clarke 2015

Pool-Seq

14 < MAC 3

3 > MAC 3

17

1

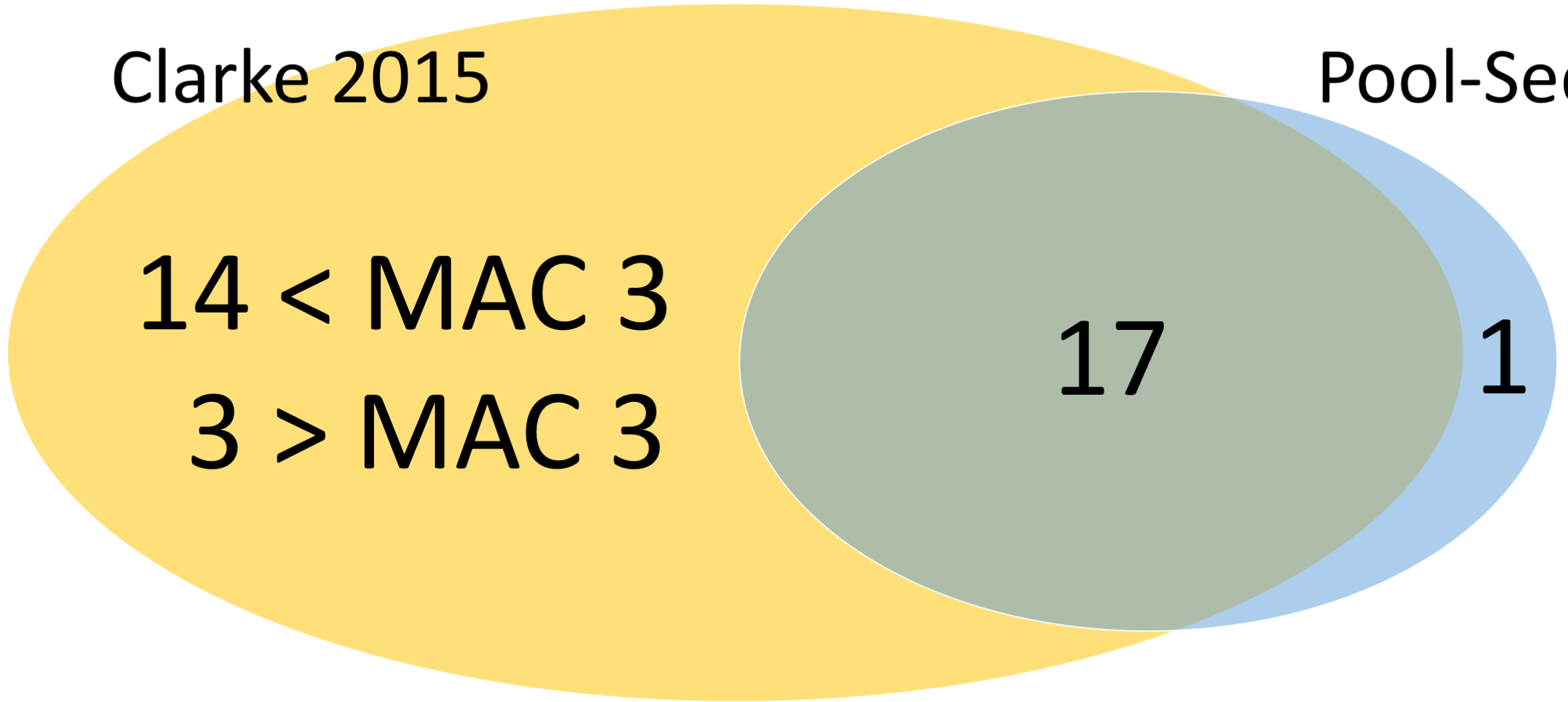

Supplement: Supplemental Information 1 — Minor allele count (MAC) are listed separately for the Clarke study because MAC < 3 were filtered out for pool-seq data. [file peerj-08-10186-s001.pdf]

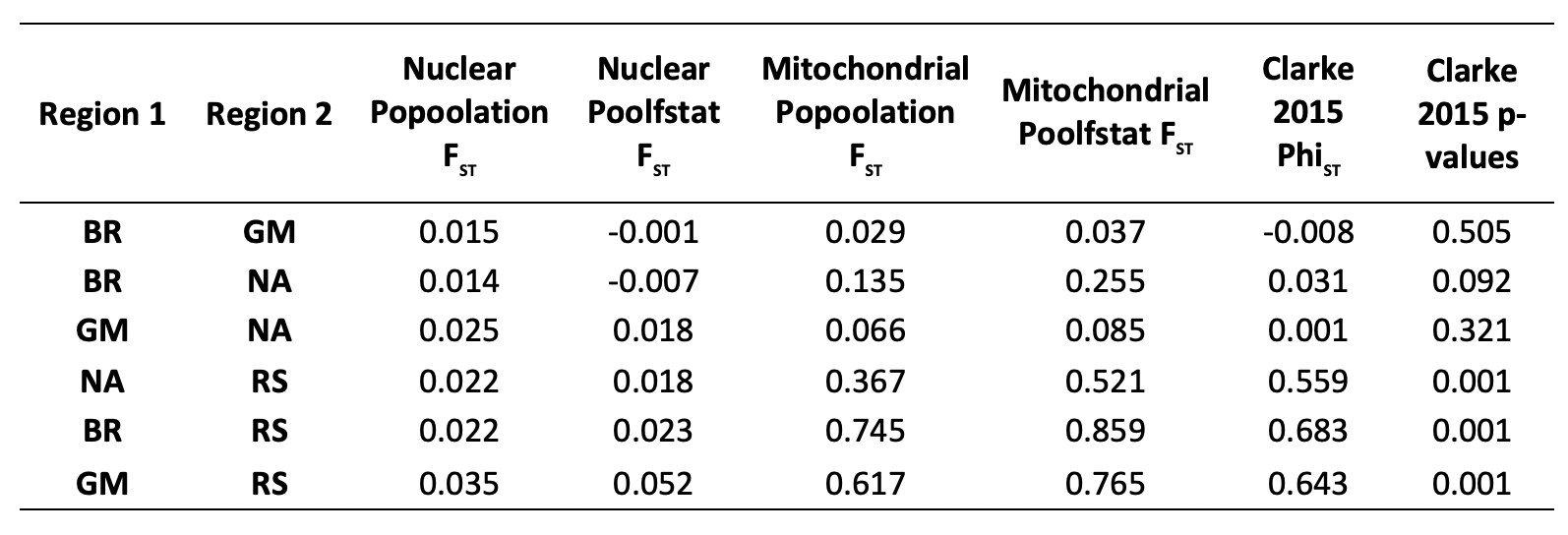

Supplement: Supplemental Information 3 — Comparison of FST matrices produced among the Popoolation approach and Poolfstat approach shows strong correlation (Mantel r=0.991 for mitochondrial and r=0.978 for nuclear data, p < 0.05). This indicates, at least for this study, the difference in the analytical approaches does not make an overall difference in this study’s conclusions. Additionally poolfstat is not yet capable of calculating p-values for confidence, so for the sake of this study only FST values calculated from Popoolation are used. This may need to be re-evaluated for studies with more pooled regions examined or larger sample sizes. Regional Abbreviations: GM = Gulf of Mexico, NA = North Atlantic, BR = Brazil, RS = Red Sea. [file peerj-08-10186-s003.docx]
