# Supplementary material for: Genomics versus mtDNA for resolving stock structure in the silky shark (Carcharhinus falciformis)
Source: PeerJ. 2020 Oct 21;8:e10186. doi: 10.7717/peerj.10186 (PMC7585369; doi:10.7717/peerj.10186)
Supplement: Supplemental Information 2 [file peerj-08-10186-s002.docx]

**Table S1.** SNP filtering parameters as found in AssessPool.

| AssessPool Filter | Selected for this study | Explanation |
| --- | --- | --- |
| min.pool.number | 2 | Minimum number of pools in which a SNP needs to be present in to be included. Using 2 can quantify small differences between each pairwise comparison. |
| min.quality.score | 20 | Drops SNPs with Phred quality scores lower than this threshold. For this data set 20 was still conservative without over filtering the data. |
| min.depth.threshold | 30 | Drops SNPs with less than 30 coverage, which is about the pool size so it assumes good coverage of all individuals in the pool as recommended by Schlötterer (2014). |
| max.missing | 3 | Maximum amount of dropped genotypes due to low coverage for a SNP to be included. 3 was selected so SNPs had to be present in at least 1 pool. |
| max.allele.length | 10 | Drops SNPs with an allele length greater than this threshold. 10 drops large indels from the data set. |
| quality.depth.ratio | 0.25 | Drops SNPS with a quality score:depth ratio lower than this threshold which removes low quality, high depth SNPs indicating loci that were likely over-grouped. |
| max.mean.depth.threshold | 1000 | This remove paralogs and multicopy loci, assessPool has graphical output to help select this value based on the dataset. |
