# Supplementary material for: Genomics versus mtDNA for resolving stock structure in the silky shark (Carcharhinus falciformis)
Source: PeerJ. 2020 Oct 21;8:e10186. doi: 10.7717/peerj.10186 (PMC7585369; doi:10.7717/peerj.10186)
Supplement: Supplemental Information 4 [file peerj-08-10186-s004.docx]

dDocent version 2.2.15 was edited at line 397 to this code:

freebayes -b split.$1.bam -t mapped.$1.bed -v raw.$1.vcf -f reference.fasta -m 5 -q 5 -E 3 -min-repeat-entropy 1 -V --pooled-continuous
